# Salinomycin Potentiates the Cytotoxic Effects of TRAIL on Glioblastoma Cell Lines

**DOI:** 10.1371/journal.pone.0094438

**Published:** 2014-04-16

**Authors:** Alessia Calzolari, Ernestina Saulle, Maria Laura De Angelis, Luca Pasquini, Alessandra Boe, Federica Pelacchi, Lucia Ricci-Vitiani, Marta Baiocchi, Ugo Testa

**Affiliations:** Department of Hematology, Oncology and Molecular Medicine, Istituto Superiore di Sanità, Rome, Italy; University of Navarra, Spain

## Abstract

Tumor necrosis factor-related apoptosis-inducing ligand (TRAIL) has been reported to exhibit therapeutic activity in cancer. However, many tumors remain resistant to treatment with TRAIL. Therefore, small molecules that potentiate the cytotoxic effects of TRAIL could be used for combinatorial therapy. Here we found that the ionophore antibiotic salinomycin acts in synergism with TRAIL, enhancing TRAIL-induced apoptosis in glioma cells. Treatment with low doses of salinomycin in combination with TRAIL augmented the activation of caspase-3 and increased TRAIL-R2 cell surface expression. TRAIL-R2 upmodulation was required for mediating the stimulatory effect of salinomycin on TRAIL-mediated apoptosis, since it was abrogated by siRNA-mediated TRAIL-R2 knockdown. Salinomycin in synergism with TRAIL exerts a marked anti-tumor effect in nude mice xenografted with human glioblastoma cells. Our results suggest that the combination of TRAIL and salinomycin may be a useful tool to overcome TRAIL resistance in glioma cells and may represent a potential drug for treatment of these tumors. Importantly, salinomycin+TRAIL were able to induce cell death of well-defined glioblastoma stem-like lines.

## Introduction

Glioblastoma (GBM) is the most common and lethal brain tumor and current standard therapies including surgery, chemotherapy and radiation provide no curative treatments. Thus, developing of new treatment strategies remains as necessary as ever [1].

A particularly promising novel therapeutic approach for GBM is the reactivation of apoptosis by treatment with members of the tumor necrosis factor (TNF) family, of which the TNF-related apoptosis-inducing ligand (TRAIL) holds the greatest appeal [2]. TRAIL exerts its function by binding its membrane receptors, designated TRAIL-R1/DR4, TRAIL-R2/DR5, TRAIL-R3/DcR1 and TRAIL-R4/DcR2. Of these receptors, only TRAIL-R1 and TRAIL-R2 transmit the apoptotic signal, while TRAIL-R3 and TRAIL-R4 are thought to function as decoy receptors that modulate TRAIL sensitivity [2].

TRAIL is a promising cancer drug because it induces apoptosis almost specifically in tumor cells with minimal or no effect on normal cells [3], [4]. Unfortunately, a considerable number of cancer cell types, including glioblastoma, have been found to be resistant to the apoptotic stimuli of TRAIL. Therefore, the combination of TRAIL with small molecules has been investigated as a strategy to potentiate TRAIL cytotoxicity by the sensitization of TRAIL-resistant cancer cells [5].

Salinomycin is a carboxylic polyether ionophore isolated from *Streptomyces albus* [6] and commercially used as a coccidiostat for poultry and a growth promoter for ruminants. Recently, Gupta *et al.* have shown in a high-throughput screen that salinomycin was a 100 times more effective killer of breast cancer stem cells than paclitaxel, a commonly used breast cancer chemotherapeutic drug [7]. Although the mechanism of anticancer activity of salinomycin is largely unknown, it appears that it might induce terminal epithelial differentiation accompanied by cell cycle arrest rather than trigger cytotoxicity [7]. The discovery of antineoplastic effects of salinomycin by Gupta *et al*. has stimulated an intensive research to investigate these new properties of the molecule and its potential clinical use for the treatment of cancer [8]. Recent studies have shown that salinomycin was able to block the proliferation of cancer stem cells of gastrointestinal stromal tumors and also to increase their sensitivity to the Kit/PDGFR inhibitor imatinib [9]. Furthermore, salinomycin was reported to inhibit cancer stem cells derived from human lung adenocarcinoma A549 cells and decreased in these cells the expression of stem cell markers [10]. Finally, salinomycin was able to target and to deplete mesenchymal-like subpopulations, within squamous cell carcinomas [11].

The mechanism through which salinomycin affects cancer cell proliferation is largely unknown. A recent report showed that salinomycin induces apoptosis in human cancer cells through a caspase-independent mechanism [12].

Other studies have shown that salinomycin is a p-glycoprotein (Multi Drug Resistance 1) inhibitor [13] and overcomes p-glycoprotein-mediated multidrug and apoptosis resistance [13], [14]. In line with these observations, salinomycin was shown to sensitize various types of cancer cells to doxorubicin and etoposide [15].

Very recently, it was shown that salinomycin acts as a very potent inhibitor of the Wnt signalling cascade and, through this mechanism, affected many Wnt target genes, including cyclin D1, fibronectin, LEF1 and depressed also LRP6 (Wnt co-receptor, lipoprotein receptor related protein 6) levels inducing its degradation [16]. Wnt/β-catenin signalling pathway seems to be responsible also for the inhibitory effect exerted by salinomycin on osteosarcoma tumor stem cells [17].

The effects of salinomycin on brain tumor cells do not have been explored. In this context, a recent study, using the glioma DBTRG-05MG cell line, showed that tumor cells surviving to hydroxyurea or aphidicolin are slowly depleted by treatment with salinomycin [18].

In the present study, we investigated the effects of TRAIL, salinomycin and the combination of both agents in GBM cell lines. The results demonstrated that salinomycin enhanced TRAIL-induced apoptosis, mainly by up-regulating the expression of TRAIL-R2.

## Results

### Antiproliferative effects of salinomycin and TRAIL on GBM cell lines

**Figure 1:**
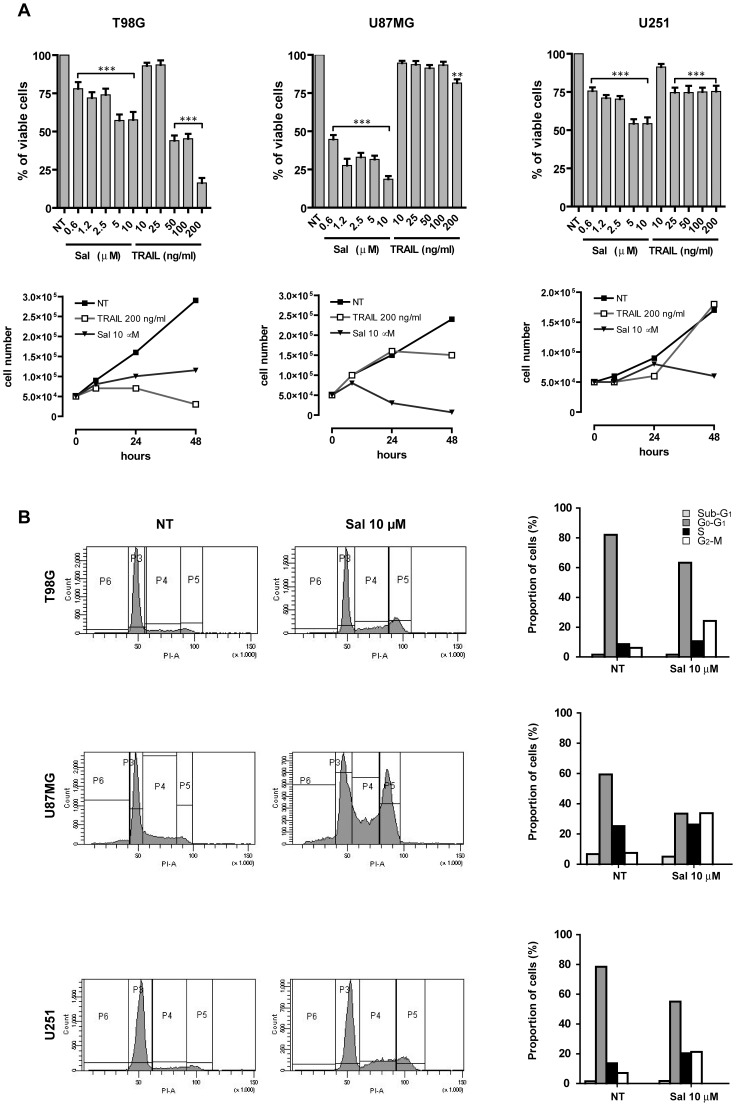
Effect of salinomycin and recombinant human TRAIL on viability and cell cycling of GBM cell lines. (A) GBM cells T98G, U87MG and U251 have been plated either in the absence (not treated, NT) or in the presence of increasing concentrations of either salinomycin (Sal) or TRAIL and the percentage of viable cells after 48 h of treatment (*top panel*) and the number of living cells at 24 and 48 h (*bottom panel*) have been determined. Results reported in the top panel represent mean values ± SEM observed in three separate experiments. ** and *** different from control (NT) at significance level *p*<0.01 and *p*<0.001, respectively. Results reported in the bottom panel represent mean values observed in three separate experiments. (B) Representative flow cytometric analysis of cell cycle of GBM cells grown for 48 h either in the absence (NT) or in the presence of 10 µM salinomycin (Sal) (*left panel*). Proportion of cells into the various phases of the cell cycle, subdivided into three fractions G0–G1, S and G2–M; the proportion of apoptotic sub-G1 cells is also reported (*right panel*). The data reported represent the mean values observed in three separate experiments.

We have evaluated the effect of salinomycin on the proliferation of three human glioblastoma cell lines, U87MG, U251 and T98G. Dose-response experiments carried out on cells exposed to increasing concentrations of salinomycin (from 0.6 to 10 µM) showed a decrease of viable cells, marked in U87MG cells and moderate in T98G and U251 cells, respectively (Fig. 1A). Maximal effects were observed at a salinomycin concentration corresponding to 5–10 µM. In parallel, we have evaluated the sensitivity of these cells to increasing doses of TRAIL, showing that only T98G, but not U87MG and U251 cells were sensitive to the cytotoxic effects of this death ligand (Fig. 1A, top panel). Cell growth experiments carried out during 48 h exposure to these drugs confirmed the results showing that salinomycin clearly inhibited the growth of all these three cell lines, its effect being particularly pronounced in U87MG cells (Fig. 1A, bottom panel).

Previous studies on other cancer models have reported a consistent anti-proliferative activity of salinomycin. To this end we have tried to analyse the mechanisms through which salinomycin may exert its inhibitory effect on cell growth. Thus, we have first explored the effect of salinomycin on cell cycle. Flow cytometry analysis of the cell cycle on the salinomycin-sensitive cell line T98G showed that this drug induced a significant accumulation of tumor cells into S and G2/M fractions (Fig. 1B). A similar, but less pronounced, effect of salinomycin has been observed also in the other two cell lines, U87MG and U251 (Fig. 1B).

### Salinomycin enhances TRAIL-induced apoptosis in GBM cell lines

**Figure 2:**
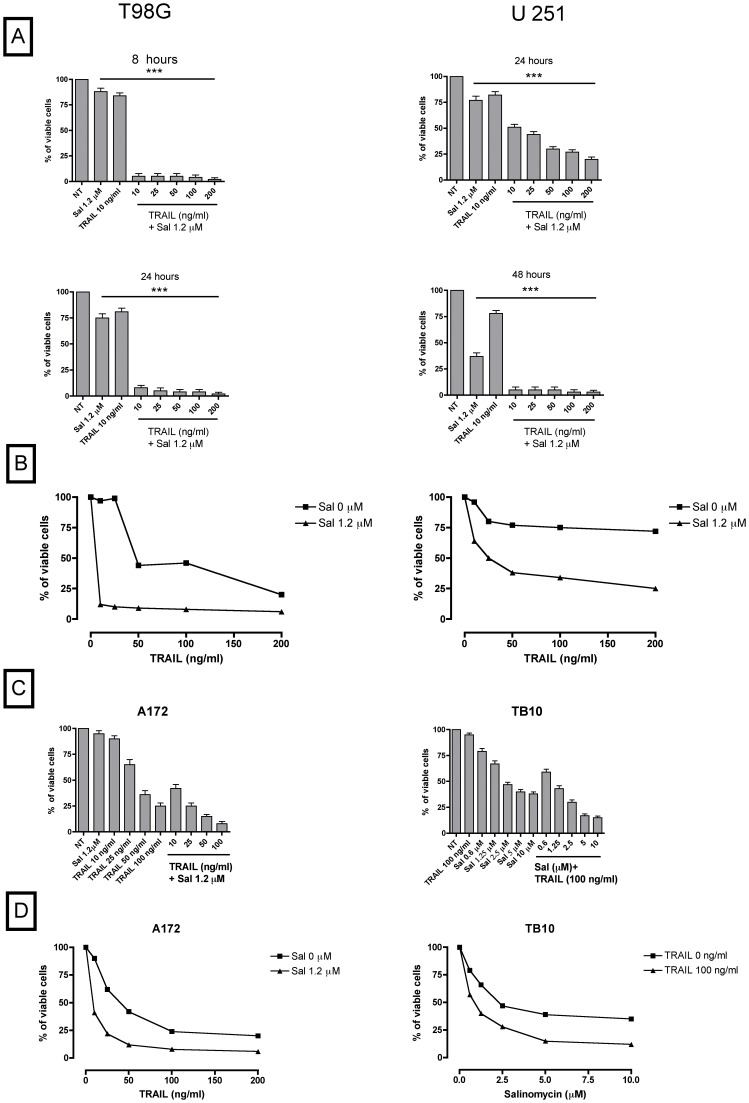
Synergistic induction of cell death by salinomycin and TRAIL. (A) T98G and U251 cell lines have been grown for various periods of time (indicated in each panel) in the absence (NT) or in the presence of 1.2 µM salinomycin (Sal) added alone or in combination with increasing doses of TRAIL (from 10 to 200 ng/ml). At the end of the incubation the percentage of viable cells was determined by trypan blue exclusion test. The data represent the mean ± SEM values observed in three separate experiments. *** different from control (NT) at significance level *p*<0.001. (B) T98G and U251 cells have been grown in the presence of increasing concentrations of TRAIL (from 10 to 200 ng/ml) either in the absence (Sal 0) or in the presence of 1.2 µM salinomycin (Sal 1.2 µM). The results are plotted assuming the 100% value of cell vitality for either Sal 0 or Sal 1.2 µM, in the absence of TRAIL. The data represent mean values observed in three separate experiments. * and ** different from control (NT) at significance level *p*<0.05 and *p*<0.01, respectively. (C) A172 cell line has been grown for 48 h in the absence (NT) or in the presence of increasing concentrations of TRAIL (from 10 to 100 ng/ml), 1.2 µM Salinomycin (Sal) added alone or in the presence of increasing TRAIL concentrations. TB10 cell line has been grown in the absence (NT) or in the presence of increasing concentrations of Salinomycin (Sal, from 0.6 to 10 µM) or in the presence of 50 ng/ml TRAIL (TRAIL) added alone or in combination with increasing concentrations of Salinomycin. At the end of the incubation period the percentage of viable cells was determined by trypan blue exclusion test. The data represent the mean±SEM values observed in three separate experiments. (D) The A172 and TB10 cells were grown as reported in C and the results were plotted assuming the 100% value of cell viability for either Sal 0 or Sal 1.2 µM (A172 cells) or for either TRAIL 0 or TRAIL 50 ng/ml (TB10 cells). The data represent mean values observed in three separate experiments.

To evaluate the effect of the contemporaneous addition of salinomycin and TRAIL in GBM cells, T98G and U251 cell lines were treated with a low concentration (i.e., 1.2 µM) of salinomycin, not suitable for inducing a significant reduction of viable cells and in the absence or in the presence of increasing concentrations of TRAIL. Interestingly, the addition of TRAIL, even at low concentrations, i.e., at 10 ng/ml, caused a marked reduction of viable cells, thus providing evidence that salinomycin and TRAIL strongly synergize to induce glioblastoma cell death (Fig. 2A). To provide a more direct evidence that salinomycin and TRAIL synergize in inducing cell death of glioblastoma cells, we plotted the cell viability data giving the 100% value both at measurements performed either in the absence (0) or in the presence of 1.2 µM salinomycin (Fig. 2B). This analysis provided evidence that in both T98G and U251 cell lines salinomycin treatment greatly sensitizes the cells to the cytotoxic effects of TRAIL.

To provide additional evidence about the capacity of salinomycin to enhance the sensitivity of glioblastoma cells to TRAIL, we have explored the effects of this drug on other glioblastoma cell lines. Thus, we have studied two additional glioblastoma cell lines, A172 sensitive to TRAIL-mediated cytotoxicity and TB10 resistant to TRAIL. In both these cell lines there was clear evidence that salinomycin enhances the sensitivity to TRAIL, as shown by experiments similar to those performed for the other glioblastoma cell lines (Fig. 2C and 2D).

According to these observations we conclude that salinomycin and TRAIL synergize in inducing cell death of glioblastoma cells.

**Figure 3:**
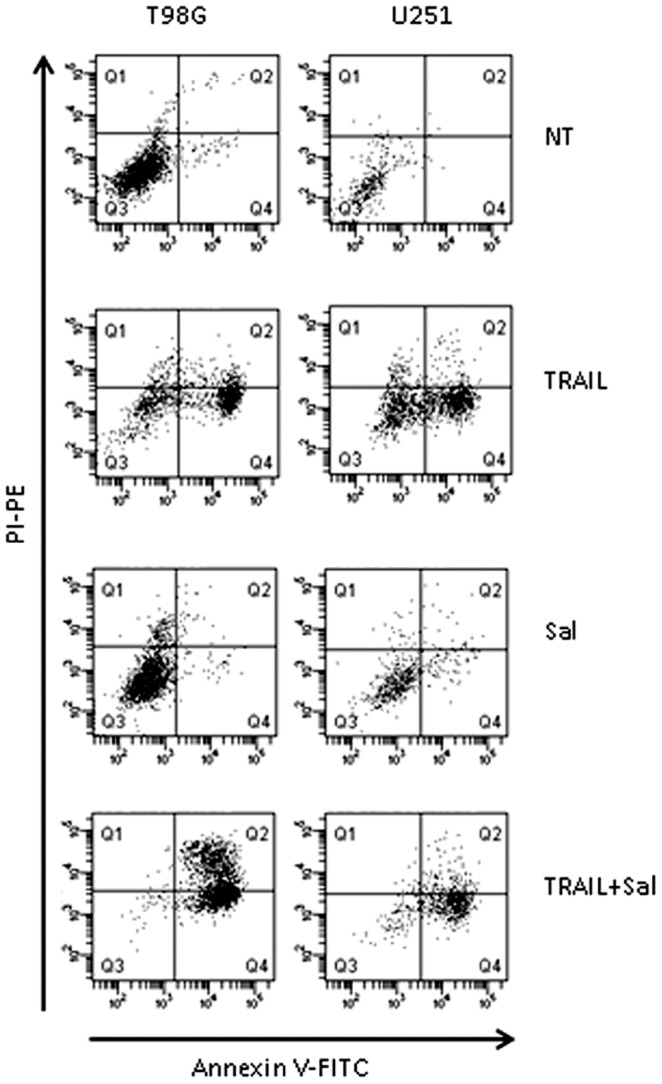
Effect of salinomycin and TRAIL added alone or in combination on the induction of apoptosis. T98G and U251 cells were incubated for 24(NT) or in the presence of salinomycin (10 µM), TRAIL (50 ng/ml) or salinomycin+TRAIL (at the above concentrations). The induction of apoptosis was evaluated by Annexin V-FITC and propidium iodide (PI) staining.

Since the cytotoxic effect of TRAIL is related to the induction of apoptosis, we have examined the flow cytometry pattern of Annexin V-FITC stained cells after treatment with TRAIL, salinomycin and their combination (Fig. 3). Cells positive for Annexin V-FITC and negative for propidium iodide (PI) are in early stage of apoptosis as shown in Q4 quadrant, while cells positive for both Annexin V-FITC and PI are in the late stage of apoptosis or necrosis as shown in Q2 quadrant. Thus, the degree of apoptosis correlates with the amount of positive Annexin V-FITC cells. The results clearly showed that salinomycin-treated cells were not able to bind Annexin V-FITC and therefore to induce apoptosis. In contrast, the proportion of Annexin V-FITC positive cells increased in response to TRAIL and even more after the co-treatment with TRAIL and salinomycin, demonstrating that salinomycin potentiates the apoptotic effect of TRAIL, both in T98G and U251 cells (Fig. 3).

**Figure 4:**
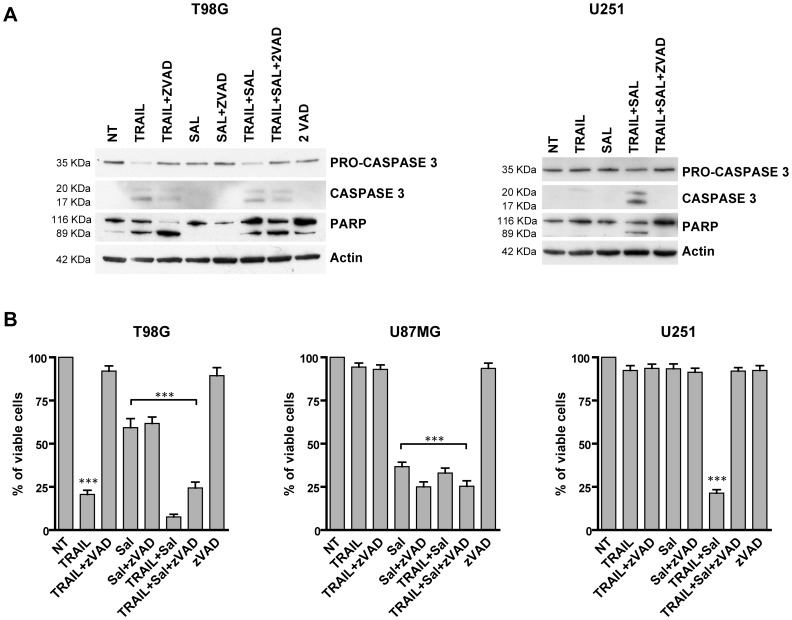
Mechanism of cell death induced by salinomycin+TRAIL. (A) Western blot analysis of cellular extracts derived from T98G and U251 cells incubated for 24 h either in the absence (NT) or in the presence of either salinomycin 10 µM, TRAIL (50 ng/ml) or salinomycin+TRAIL (at the above concentrations) or salinomycin+TRAIL+the caspase inhibitor zVADfmk (40 µM). The cell extracts were first run on SDS-PAGE, transferred to nitrocellulose membranes and blotted with either anti-human caspase-3, anti-human PARP or anti beta-actin. One representative experiment out of three performed is shown. (B) T98G, U87MG and U251 cells have been grown for 24 h in the different experimental conditions reported in the figure and then analysed for cell vitality. The proportion of viable cells is reported (mean values ± SEM observed in three separate experiments). *** different from control (NT) at significance level *p*<0.001.

We have then investigated whether the cytotoxic effect of salinomycin could be ascribed to a biochemical pathway involving caspase activation. To this end we have performed two types of experiments. First, we have evaluated the effect of salinomycin on caspase-3 activation by Western blot analysis. Salinomycin alone failed to induce any significant caspase-3 activation in both T98G and U251 cells. Combining salinomycin with TRAIL resulted in a clear caspase-3 activation as evidenced by the formation of cleavage fragments of pro-caspase-3, corresponding to activated caspase-3, and by the poly-ADP-ribose-polymerase (PARP) cleavage (Fig. 4A). Next, we have tried to inhibit salinomycin-induced cytotoxicity using the pan-caspase inhibitor zVADfmk. The addition of zVADfmk was unable to protect T98G cells from caspase-3 activation and PARP cleavage induced by salinomycin+TRAIL treatment (Fig. 4A). In contrast, zVADfmk fully protected from salinomycin+TRAIL-mediated caspase-3 activation and PARP cleavage in U251 cells (Fig. 4A). Cell growth experiments carried out during 24 h exposure to salinomycin, TRAIL and the combination of both in GBM cells preincubated with zVADfmk confirmed that the caspase inhibitor fully suppressed the cell death induced by co-treatment with salinomycin and TRAIL in U251 cells, but not in T98G and U87MG cells (Fig. 4B).

### Salinomycin enhanced TRAIL-induced apoptosis through TRAIL-R2 upregulation on the cell surface

**Figure 5:**
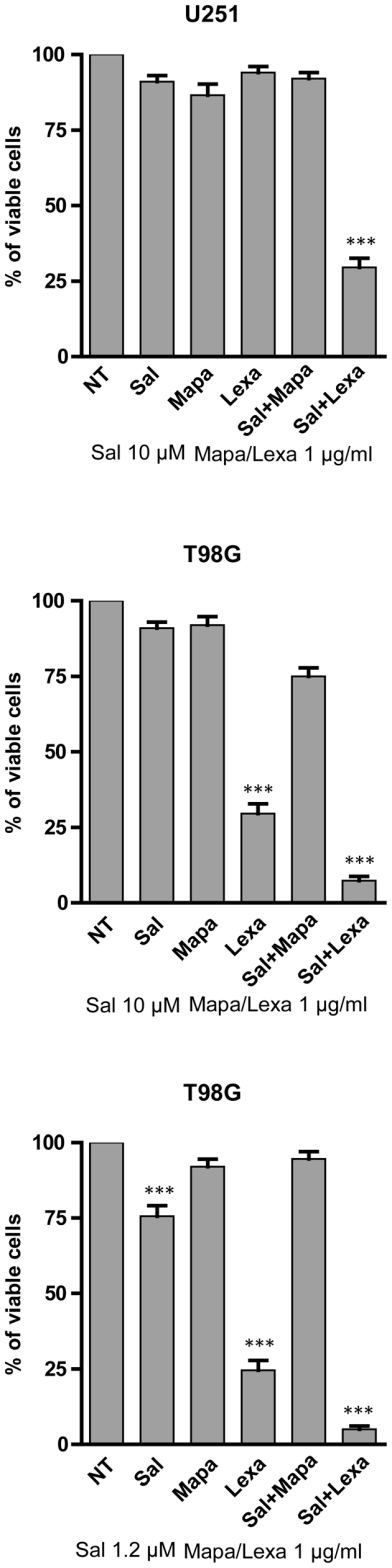
Effect of agonistic anti-TRAIL-R1 (Mapatumamab) or anti-TRAIL-R2 (Lexatumamab) mAbs on the induction of cell death of T98G, U87MG and U251 cell lines. The cells were incubated for 24(Sal), Mapatumamab (Mapa) and Lexatumamab (Lexa) and then analysed for cell vitality. The results represent the mean values ± SEM observed in three separate experiments. *** different from control (NT) at significance level *p*<0.001.

**Figure 6:**
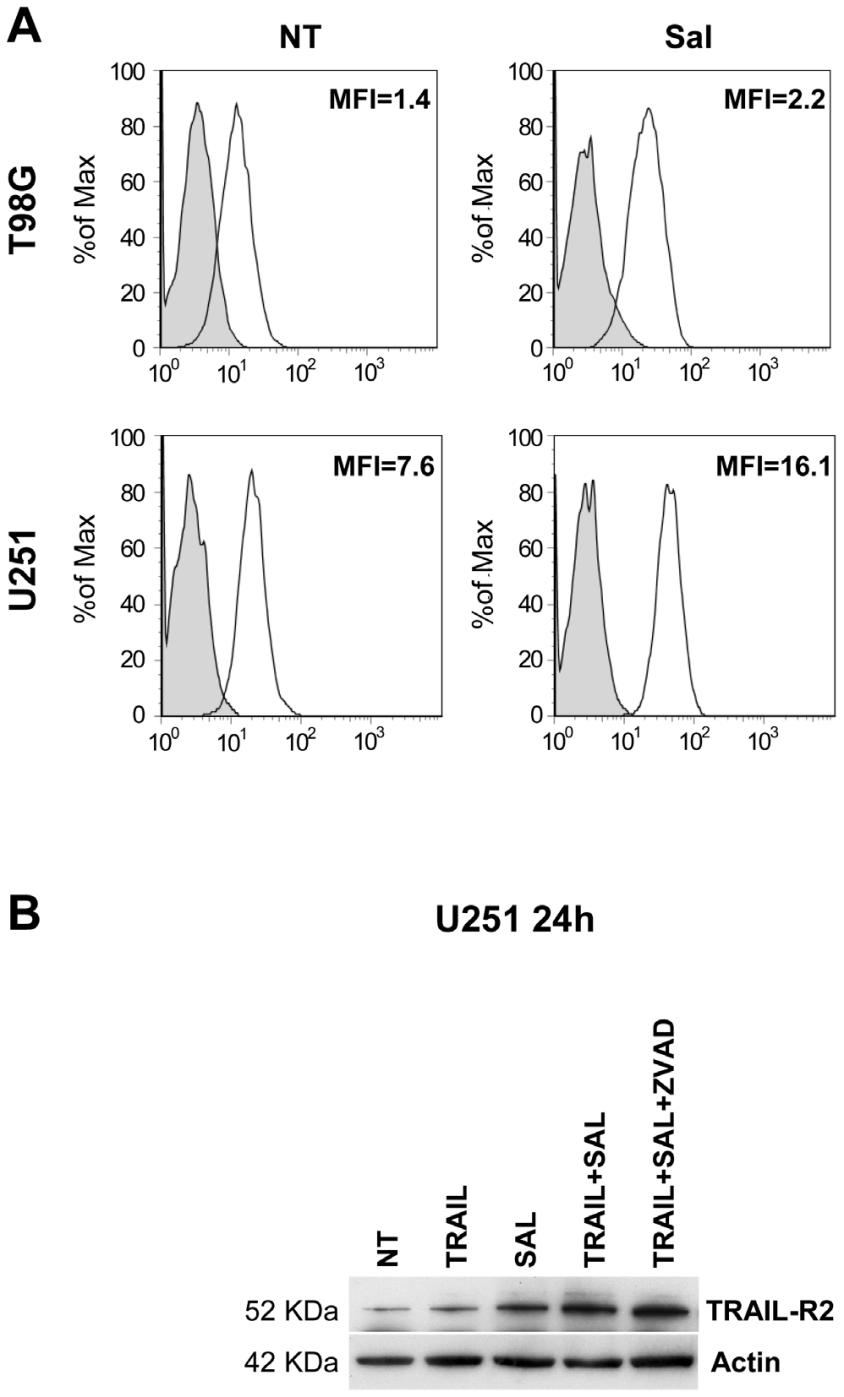
Effect of salinomycin on TRAIL-R2 expression. (A)Flow cytometric analysis of TRAIL-R2 expression of T98G and U251 cells grown for 24 h in the absence (NT) or in the presence of 10 µM salinomycin (Sal). (B) Western blot showing protein levels of TRAIL-R2 in U251 cells after treatment for the indicated time with TRAIL, salinomycin (Sal), TRAIL+salinomycin (TRAIL+Sal) and TRAIL+salinomycin+zVAD.

Salinomycin could potentiate TRAIL-mediated cytotoxicity on GBM cells acting through different mechanisms. One of these mechanisms could be related to a stimulation of either TRAIL-R1 or TRAIL-R2, the two receptors that mediate the cytotoxic effects of TRAIL, activating the extrinsic cell death pathway. Thus, in a first set of experiments we evaluated the effect of agonistic anti-TRAIL-R1 (Mapatumamab) and anti-TRAIL-R2 (Lexatumamab) on cell growth. Lexatumamab, but not Mapatumamab, when added alone induced cell death in the TRAIL sensitive T98G cell line; in contrast, in the U251 cell line, resistant to TRAIL, Lexatumamab failed to induce cell death. The combination of Lexatumamab and salinomycin, even at low doses, resulted in a very pronounced cytotoxicity (Fig. 5). Given these results it seemed of interest to evaluate a possible modulatory effect of salinomycin treatment on TRAIL-R2 expression in GBM cell lines. Flow cytometry and Western blot experiments clearly showed that salinomycin treatment induced an upmodulation of TRAIL-R2 expression in T98G and U251 cells (Fig. 6).

**Figure 7:**
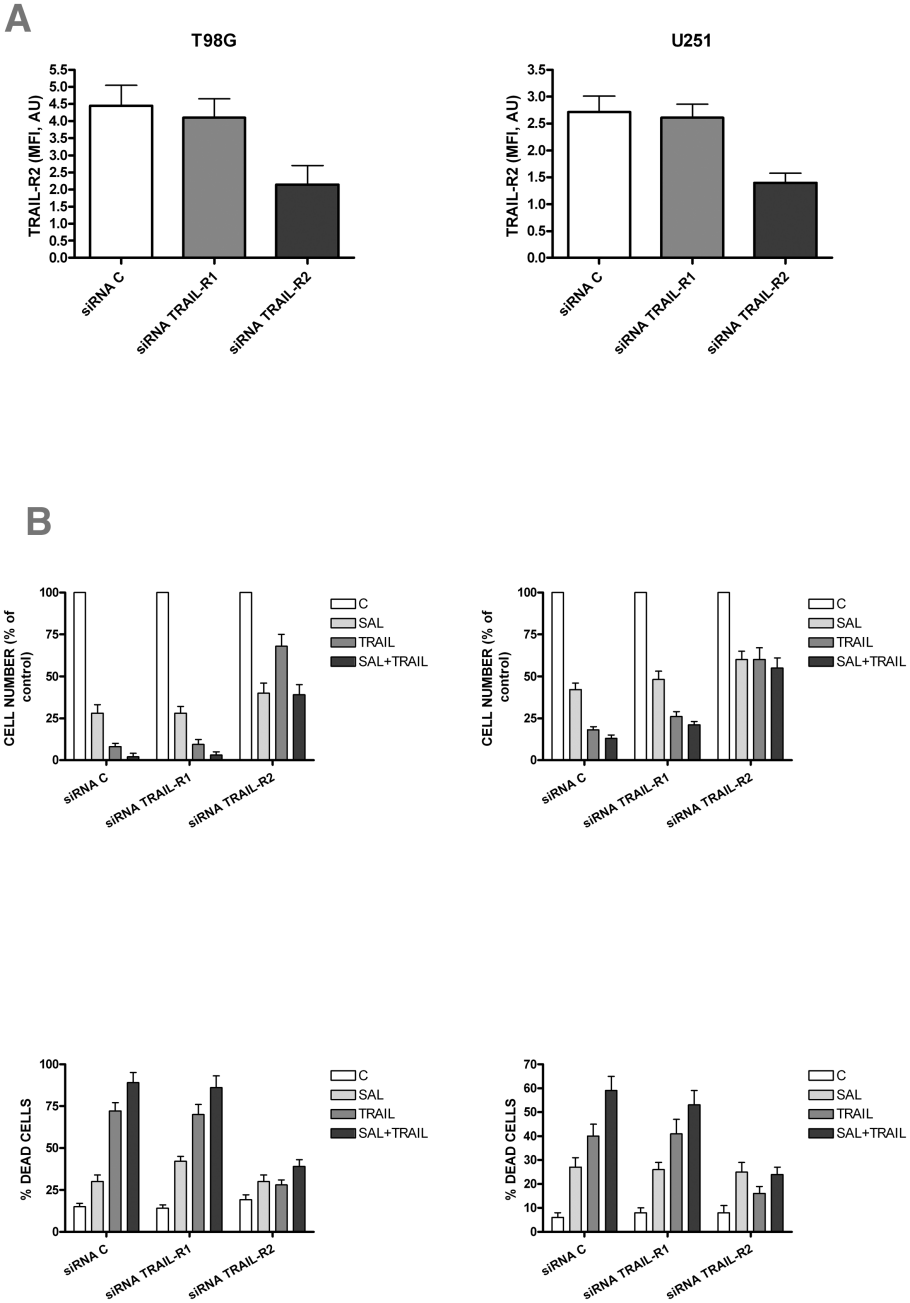
Effect of silencing of RNA encoding TRAIL-R2 on the cell growth inhibition and induction of cell death induced by salinomycin or TRAIL added alone or in combination. T98G (left panels) and U251 (right panels) cells have been incubated for 72 h either with siRNA C or siRNA TRAIL-R1 or siRNA TRAIL-R2 and then incubated for additional 24 hours either in the absence of additives (Control, C) or in the presence of either salinomycin (10 µM) or TRAIL (50 ng/ml) or both compounds at the above doses. At the end of this time the cells have been recovered and evaluated for TRAIL-R2 expression (top panels) by flow cytometry or for the cell survival (middle panels) by cell counting after trypan blue staining or for the evaluation of cell death (assayed by flow cytometry after labelling with annexin V and propidium iodide). The data reported in the figure represent mean values ± SEM observed in three separate experiments. The statistical analysis of the data showed: in top panels a very significant difference between siRNA TRAIL-R2 and si RNA C and siRNA TRAIL-R1 (for both p<0.01); in middle and bottom panels a very significant difference for both TRAIL and TRAIL+Salinomycin-treated samples between siRNA TRAIL-R2 and siRNA C (p<0.01) and siRNA TRAIL-R1 (p<0.01).

In order to provide direct evidence that the stimulatory effect of salinomycin on TRAIL-mediated apoptosis could be at least in part mediated through TRAIL-R2 upmodulation, TRAIL-R2 expression was knockdown using specific siRNA. To this end, T98G and U251 cells have been treated with a siRNA specific for TRAIL-R2 or with a control scrambled siRNA, or with a siRNA specific for TRAIL-R1. Cells treated with these various siRNAs were then incubated for 24 hours with no additives (control) or with either TRAIL (50 ng/nl) or with salinomycin (10 µM) or both agents at the above doses. The results of these experiments (Fig. 7) have shown that in T98G and U251 cells: (i) siRNA TRAIL-R2, but not control siRNA or siRNA TRAIL-R1, significantly reduced TRAIL-R2 expression, as assessed by flow cytometry experiments (Fig. 7, top panels); siRNA TRAIL-R2, but not siRNA TRAIL-R1 or control siRNA, was able to consistently reduce the inhibition of cell vitality or the increase in apoptotic cells induced by TRAIL or salinomycin+TRAIL, but not by salinomycin alone (Fig. 7, middle and bottom panels). These observations support a functional role for TRAIL-R2 in mediating the stimulatory effect exerted by salinomycin on TRAIL-mediated apoptosis of glioblastoma cell lines.

### Effects of salinomycin treatment on caspase-8 activation and mitochondrial membrane depolarization

**Figure 8:**
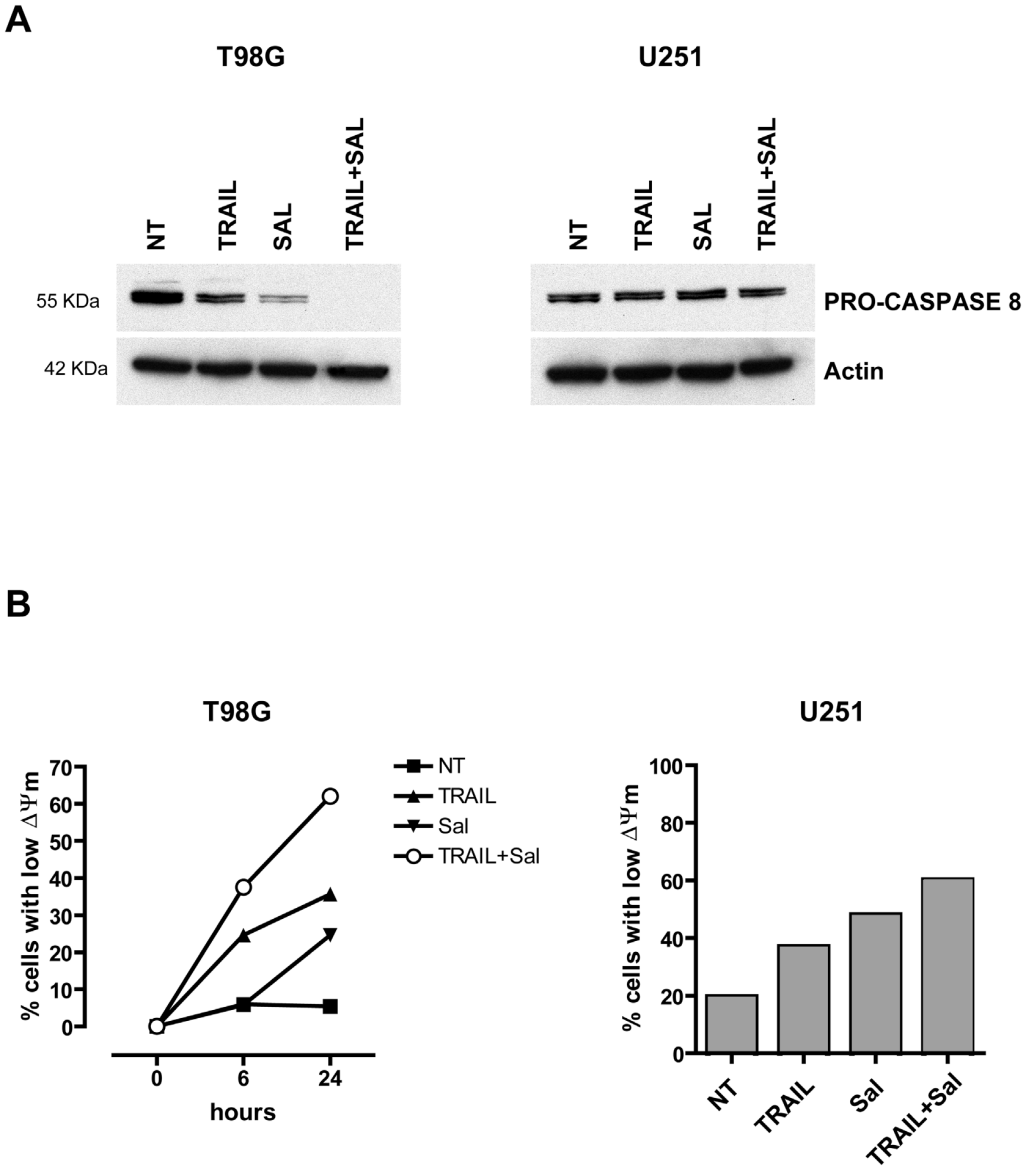
Effect of salinomycin and TRAIL added alone or in combination on caspase-8 activation and mitochondrial function. (A) Western blot analysis of cellular extracts derived from T98G and U251 cells incubated for 8 h and 24 h respectively, either in the absence (NT) or in the presence of salinomycin (10 µM), TRAIL (50 ng/ml) or salinomycin+TRAIL (at the above concentrations). The cell extracts were first run on SDS-PAGE, transferred to nitrocellulose membranes and blotted with either anti-human caspase-8 or anti beta-actin. One representative experiment out of three performed is shown. (B) Analysis of the mitochondrial membrane potential (Ψm) in T98G and U251 cells grown in the various conditions reported in the figure for 8 h and 24 h (T98G) or 48 h (U251). The proportion of cells exhibiting low Ψm is reported.

In a subsequent set of experiments we have explored the functional role of caspase-8 activation in the capacity of salinomycin to potentiate the antitumor effects of TRAIL. Western blot analysis showed that salinomycin elicited a marked increase in caspase-8 activation, as evidenced on the basis of the decrease of pro-caspase-8 levels in TRAIL-sensitive T98G, but not in U251 cells (Fig. 8A).

Since early mitochondrial perturbations are observed during the induction of apoptosis and other types of cell death [19], we have evaluated the loss of mitochondrial membrane potential (ΔΨm) using the JC-1 dye. JC-1 is cationic dye that exhibits potential-dependent accumulation in mitochondria by fluorescence emission shift from green (∼520 nm) to red (∼590 nm). Consequently, mitochondrial depolarization is indicated by a decrease in the red–green fluorescence intensity ratio. This analysis provided clear evidence that salinomycin was able to induce a pronounced increase in the proportion of cells exhibiting a loss of ΔΨ_m_ when added alone, and this effect was potentiated by the combination of salinomycin with TRAIL (Fig. 8B). It's important to note that in the TRAIL-sensitive T98G cells, the death ligand itself induced a mitochondrial perturbation, which markedly increased due to the synergistic action of salinomycin and TRAIL (Fig. 8B, left panel). In contrast, in U251 cells, which are markedly less sensitive than T98G cells to the pro-apoptotic effects of TRAIL, the loss of ΔΨ_m_ after TRAIL and salinomycin co-treatment is probably mainly due to salinomycin action (Fig. 8B, right panel).

### Combined treatment with salinomycin and TRAIL markedly inhibits glioblastoma xenograft growth

**Figure 9:**
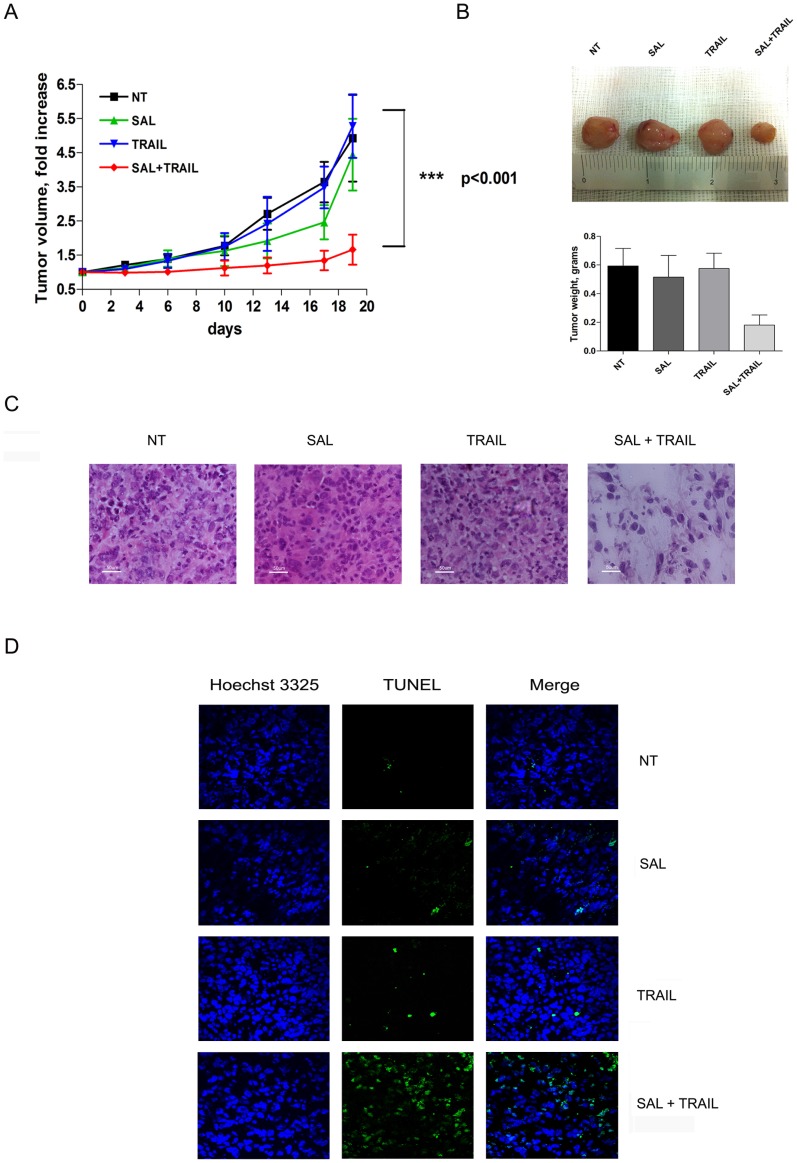
Salinomycin in combination with TRAIL markedly inhibits the growth of glioblastoma xenografts. 1×10^6^ U251 cells were injected subcutaneously into nude mice as described in Materials and Methods, and treatment was started when the tumors reached 100 mm^3^. TRAIL (5 mg/Kg) and salinomycin (200 ng/kg) were administered 3 times/week, intraperitoneally. Control mice received vehicle only, according to the same schedule. Tumor volume was measured by caliper. Tumor fold increase is reported inn the Figure. Data represent the mean of 5 tumors±SEM, and significance of the results was evaluated by ANOVA and Bonferroni post-tests, as described in the section on statistical methods. A) Tumor growth kinetic; B) Tumor weight post-sacrifice; Representative pictures of tumor mass harvested at sacrifice, for each of the four experimental subgroups (top panels) and tumor weight post-sacrifice (mean of 5±SEM); C) Tumor histological analysis of tissutal sections stained with Hematoxylin Eosin (representative pictures); D) Immunofluorescence analysis of tumor apoptotic cells detected by TUNEL reaction (representative pictures): left panels: Hoechst 3326 staining; middle panels: TUNEL staining; right panels: merged of both stainings.

To evaluate the antitumor effect of salinomycin, TRAIL, or the combined treatment *in vivo*, we generated subcutaneous xenografts by inoculating the U251 cell line in the flank nude mice. When the tumor reached a volume of about 100 mm^3^, approximately 4 weeks post inoculum, treatment was started. Tumor size and body weight were measured twice a week during treatment. As shown in Fig. 9A, treatment with TRAIL did not modify tumor growth kinetic, as compared to controls; salinomycin induced a limited delay in tumor growth, not reaching statistical significance. Finally, combined treatment with salinomycinnand TRAIL elicited a marked inhibitory effect on xenograft development (p = 00.2 versus untreated mice; p = 0.03 versus TRAIL-treated mice; p = 0.045 versus salinomycin-treated mice). Tumor weight, measured after sacrifice, (Fig. 9B) confirms a pronounced and significant decrease of tumor development in mice treated with the combination of salinomycin and TRAIL, as compared to the other experimental groups. Histologic analysis of tumor sections showed a markedly decreased cellularity in Salinomycin+TRAIL-treated mice, compared to all other experimental groups (NT, Salinomycin or TRAIL) (Fig. 9C). As expected the number of apoptotic tumor cells was markedly higher in tumor-bearing mice treated with Salinomycin+TRAIL compared to all other experimental groups (Fig. 9D).

### Sensitivity of glioblastoma neurospheres to salinomycin and TRAIL

**Figure 10:**
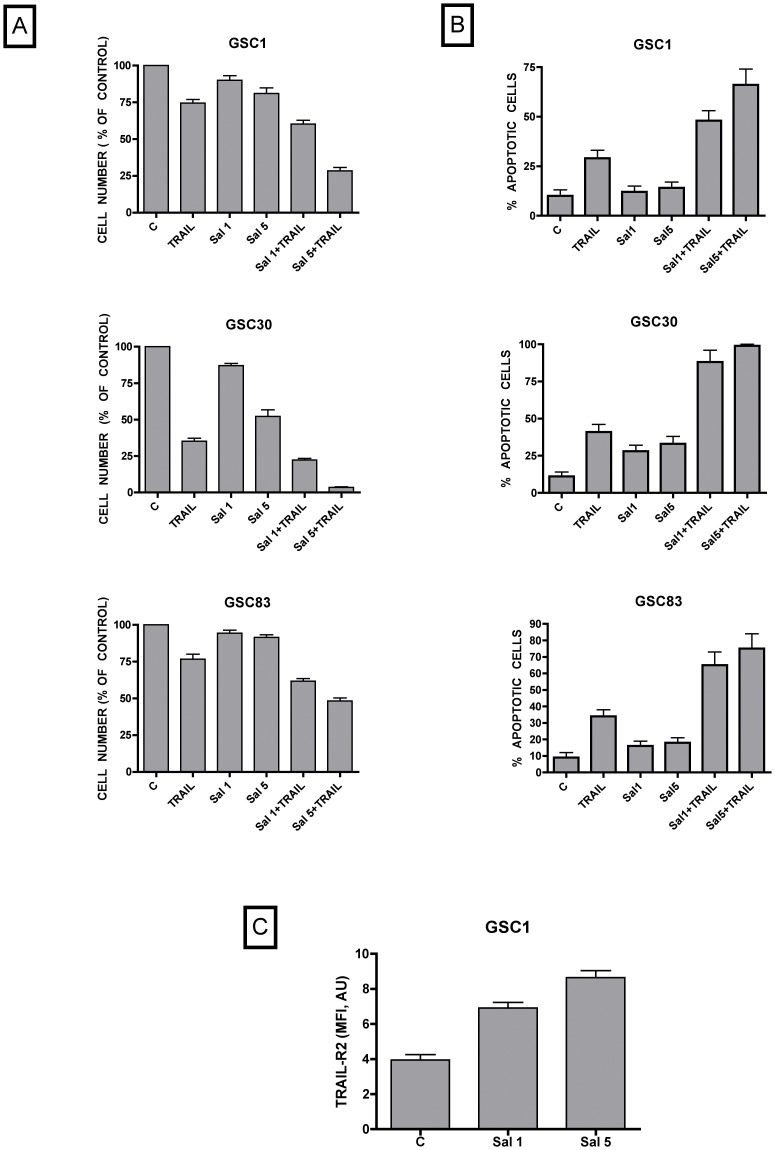
Effect of salinomycin and recombinant TRAIL on cell growth (A), cell death (B) and TRAIL-R2 (C) expression of GSC neurospheres. A and B – Glioblastoma neurosphere clones GSC1, GSC30 and GSC83 were grown for 48 hours either in the absence (C) or in the presence of either TRAIL (10 ng/ml) or Salinomycin 1 µM or Salinomycin 5 µM or Salinomycin 1 µM+TRAIL or Salinomycin 5 µM+TRAIL and the number of viable cells was determined by the quantification of cellular ATP content using the Cell Titer-Glo Luminescen Cell Viability Assay Kit (A) and the percentage of apoptotic cells by the Annexin-V binding assay (B). The results represent the mean values observed ± SEM observed in three separate experiments, each performed in duplicate. For all these treatments and for all the three neurosphere clones, the difference between the values observed for TRAIL and Salinomycin 1 µM+TRAIL or Salinomycin 5 µM+TRAIL were statistically significant (p = <0.05 or <0.01) and the values observed for Salinomycin 1 µM and Salinomycin 1 µM+TRAIL or Salinomycin 5 µM and Salinomycin 5 µM+TRAIL (p = <0.05 or <0.01) were statistically significant. (C) Flow cytometric detection of TRAIL-R2 expression in GSC1 cells grown for 24 h either in the absence or in the presence of salinomycin (1 or 5 µM). The results are expressed in terms of mean fluorescence intensity (MFI) values observed in three separate experiments (mean values±SEM). The differences between the values observed between 1 µM or 5 µM salinomycin and control are statistically significant (both p<0.01).

A subpopulation of glioblastoma stem-like cells (GSCs) that shares properties with neural precursor cells has been described, exhibiting resistance to therapy and therefore being considered responsible for the high recurrence rate of glioblastoma [20]. Therefore, given the anti-cancer stem properties of salinomycin, it seemed particularly important to evaluate a possible effect of this drug added alone or in combination with TRAIL on the growth and apoptosis of GSCs. To perform this analysis we have used glioblastoma neurosphere lines GSC1, GSC30 and GSC83, previously isolated and extensively characterized in our laboratory [21], [22]. The individual sensitivity of these glioblastoma neurosphere clones to the antiproliferative and apoptosis-inducing effects of salinomycin and TRAIL was variable (Fig. 10). However, in spite this consistent variability, the addition of salinomycin (either at 1 or 5 µM) together with TRAIL (10 ng/ml) elicited a significant increase of the inhibitory effect on cell growth and on the stimulatory effect on tumor cell apoptosis (Fig. 10).

As above mentioned, one of the mechanisms responsible for the cooperative effect induced by salinomycin on TRAIL sensitivity of glioblastoma cells lines seems to be related, at least in part, to a stimulatory effect on TRAIL-R2 expression. Thus, we have evaluated the effect of salinomycin on TRAIL-R expression of GSC neurosphere lines. All the three untreated GSC clones clearly expressed TRAIL-R2 (a higher expression was observed in GSC30 and GSC83, compared to that observed in GSC1), but not TRAIL-R1, whose expression was virtually undetectable (data not shown). Salinomycin addition clearly increased TRAIL-R2 expression, as shown by the analysis of mean fluorescence intensity values observed in three separate experiments (Fig. 10C).

## Discussion

High-grade astrocytomas, that include glioblastoma multiforme and anaplastic astrocytoma, are the most common and aggressive primary malignant brain tumors in adults. Despite improvements in overall survival with addition of temozolomide to radiation in the adjuvant setting, the prognosis of patients affected by these tumors remains particularly poor. For these reasons there is absolute need for the development of innovative therapies. In this context, particularly interesting seems the identification and evaluation of a new category of drugs of recent identification, selected according to their capacity to inhibit the growth of cancer stem cells. One of the first drugs active against cancer stem cells was salinomycin [7]. Given the peculiar properties of this antibiotic, it seemed of interest to evaluate a possible antitumor activity of this compound against glioma tumor cells. At the best of our knowledge, this is the first report describing the antitumor properties of salinomycin against malignant gliomas.

A major finding of our study is that we demonstrate for the first time that salinomycin enhances TRAIL-induced apoptosis in glioblastoma, at least in part through TRAIL-R2 upmodulation. Although many cancer cells are preferentially sensitive to TRAIL-induced apoptosis, the sensitivity of glioblastoma cells is highly variable, and most short-term primary glioblastoma cultures, as well as many glioblastoma cell lines, are TRAIL insensitive [23]. Various mechanisms operating in glioblastoma cells have been involved in TRAIL resistance of these cells: FLIP overexpression due to its stabilization mediated by PTEN loss [24]; absent/low caspase-8 and/or TRAIL-R2 expression [25]; impaired cell death signaling originated from TRAIL-R activation [26]. Recent studies have identified a new mechanism of TRAIL resistance observed in some glioblastoma cell lines (including TB10, U251 and U87MG) and mediated by overexpression of miR-21 and miR-30b/c, responsible for the targeting of caspase-3 and Tap63 mRNAs [27]. It is important to note that in the present study we have included three TRAIL-resistant cell lines, U251, TB10 and U87MG. Salinomycin was able to induce TRAIL sensitivity of U251 and TB10 cell lines, but not of U87MG. We do not know the exact molecular mechanisms responsible for U87MG cells resistance to TRAIL sensitization, but preliminary evidence suggests that it could be related to low caspase-8 expression (data not shown). Our observations are in line with a recent study showing that U87MG cells are completely resistant to TRAIL; in this study it was provided evidence that TRAIL resistance of these cells could be related to an enhanced expression of the anti-apoptotic protein Mcl-1 and agents lowering the levels of this protein restore TRAIL sensitivity [28].

TRAIL-R2 upmodulation induced by salinomycin seems to be relevant to explain the stimulatory effect of this drug on TRAIL-mediated cell death and this for several reasons. First, TRAIL-R2, but not TRAIL-R1, is the main TRAIL receptor expressed on glioblastoma cells and its activation may trigger caspase-8 cleavage and initiation of apoptosis [29]. Second, several agents that upmodulate TRAIL-R2 expression in glioblastoma cells, such as cisplatin [30], lanatoside C [31], nelfinavir [32], the protesome inhibitor SC68896 [33] restore TRAIL sensitivity of glioblastoma cells. Third, the level of TRAIL-R2 expression on glioblastoma cells positively correlates with patient's survival and alone represents an independent and significant prognostic factor for survival [34]. The suggestion that TRAIL-R2 upmodulation by salinomycin is a biochemical event essential for the stimulatory activity of this drug on TRAIL-mediated apoptosis is directly supported by experiments carried out silencing expression of TRAIL-R2 expression in glioblastoma cell lines using a specific siRNA. Thus, our experiments have shown that inhibition of TRAIL-R2 expression in T98G and U251 cells almost completely abrogated the stimulatory effect of salinomycin on TRAIL-mediated apoptosis. This observation suggests a potential use of salinomycin to bypass the intrinsic resistance of glioblastoma cells to TRAIL-mediated apoptosis. It is of interest to note that a recent study reported that monensin, a ionophore structurally related to salinomycin, overcomes TRAIL resistance in glioblastoma cell lines through TRAIL-R2 upmodulation and c-FLIP downregulation [35]. Interestingly, salinomycin induces apoptosis of cisplatin-resistant ovarian cancer cells through TRAIL-R2 upmodulation [36], thus indicating that an increased expression of this receptor may represent a general mechanism through which salinomycin enhances the sensitivity of tumor cells to the death ligand TRAIL. However, we cannot exclude that salinomycin may stimulate the sensitivity of glioblastoma stem cells to TRAIL, acting through molecular mechanisms differrent from TRAIL-R2 upmodulation. In fact, in line with this hypothesis, studies carried out in breast cancer [37] and ovarian cancer [38] cell lines have reported an inhibitory effect of salinomycin on surviving expression, dependent upon Stat3 inhibition.

However, salinomycin on its own causes a cytotoxic effect on glioblastoma cells, related to the activation of a caspase-independent cell death pathway. In fact, salinomycin-sensitive glioblastoma cell lines did not show caspase activation following salinomycin treatment; furthermore, zVAD, a pan-caspase inhibitor was unable to protect glioblastoma cells from salinomycin-induced cell death; finally, salinomycin-treated cells fail to bind annexin V, a typical feature of apoptotic cells. These findings are at variance with those reported in other tumor cell types, such as prostate cancer cells [39] and chronic lymphocytic leukemia [16], where salinomycin induces cell death by apoptosis [39]. It is of interest to note the results recently reported for another drug, lanatoside C. In fact this drug, like salinomycin, acts in glioblastoma cells as a sensitizer to TRAIL-induced cell death via TRAIL-R2 upregulation; furthermore, both lanatoside C and salinomycin, on their own, induce cell death of glioblastoma cells by a caspase-independent mechanism; finally, lanatoside C, like salinomycin, causes a decrease of mitochondrial membrane potential [40].

One important caveat for the potential clinical use of salinomycin is its severe toxicity. In fact, some incidents occurred in humans when salinomycin was accidentally ingested at relatively high doses [41]. In these cases, as well as in cases of animal poisoning, a significant neuromuscular toxicity was observed. In line with these findings, a recent study provided evidence that salinomycin at the micromolar range (1–10 µm) cause *in vitro* a cytotoxic effect on murine dorsal root ganglia neurons by means of calpain and cytochrome c-mediated caspase 9 and subsequent caspase 3 activation [42]. Therefore, in view of a possible clinical use of this antibiotic it is particularly important to identify drug combinations, allowing both to potentiate the antitumor activity of salinomycin and to decrease the concentration of this drug. The combination of salinomycin with either TRAIL or an agonistic anti-TRAIL-R2 antibody seems to fulfill both these requests. In fact, we observed a synergistic interaction between salinomycin and TRAIL, showing that salinomycin in the nanomolar range was able to greatly potentiate TRAIL-induced cell death of glioblastoma cells.

Studies carried out during the last years have shown that glioblastomas and other brain cancers contain cell hierarchies of tumor cells, with highly tumorigenic cells that display stem cell features and are capable of creating a complex tumor upon transplantations [20]. Glioblastoma stem cells are resistant to chemotherapy and radiotherapy and have also an increased capacity for invasion and angiogenesis and are, therefore, important therapeutic targets [20]. Given the scarce sensitivity of glioblastoma cells and, particularly, of glioblastoma CSCs to various anticancer agents, it seemed particularly interesting to investigate their sensitivity to salinomycin, a drug active against various types of CSCs. Through the analysis of three glioblastoma neurosphere clones we obtained evidence that they are scarcely sensitive to salinomycin and moderately sensitive to TRAIl, but are markedly inhibited in their growth and survival by the combined addition of these two agents. At the best of our knowledge, this is the first study reporting a high sensitivity of glioblastoma CSCs to the combined addition of salinomycin and TRAIL. Only a recent study reported the scarce sensitivity of two glioblastoma CSC clones to salinomycin; only the combined addition of salinomycin and a histone deacetylase inhibitor, valproic acid, elicited a moderate cytotoxic effect on these cells [43].

In conclusion, the results of the present study provide an initial set of observations suggesting a significant anti-glioblastoma activity of salinomycin in combination with TRAIL. Future studies will assess the real impact of this drug combination in malignant glioma therapy.

## Materials and Methods

### Cell culture

The glioblastoma cell lines T98G, U87MG, U251 and A172 were obtained through the courtesy of Dr R Pallini (Neurosurgery Institute, Sacre Heart Catholic University Rome, Italy). These cell lines were initially obtained from ATCC and were currently characterized for their immunophenotypic features. The TB10 cell line [44] was isolated in the laboratory of Dr R. Pallini and obtained through the courtesy of this investigator.

The human GBM cell lines U87MG, T98G, U251, A172 and TB10 were grown in DMEM medium (Gibco, Invitrogen, Milan, Italy) containing 10% fetal bovine serum (FBS). The cells were routinely checked for the presence of mycoplasma.

### Isolation, growth and analysis of glioblastoma neurospheres

Glioblastoma stem cells (GSCs) were isolated from tumor surgical specimens through mechanical dissociation of the tumor tissue and cultured at clonal density in serum-free medium supplemented with EGF and basic-FGF, as previously reported (21). This procedure was used to obtain the formation of exponentially growing neurospheres that maintain an undifferentiated state when grown in this serum-free medium (21). The tumorigenic and the main phenotypic properties of the isolated stem-like clones was previously described in detail (21). The sensitivity of three different glioma stem-like clones (GSC1, GSC30 and GSC83) to salinomycin and TRAIL was used in this study. The properties of these three neurosphere glioblastoma clones were previously reported (22).

### Reagents

Salinomycin was purchased from Sigma Co (St. Louis, USA).

Recombinant human TRAIL (rh superkiller TRAIL) was purchased from Alexis Co (Alexis, Co, Lausen, Switzerland) and recombinant human soluble TRAIL-R2 was purchased from Peprotech (Peprotech, Rocky Hill, NJ, USA). The agonistic monoclonal antibodies to TRAIL-R1 (Mapatumamab) and TRAIL-R2 (Lexatumamab) are fully human antibodies of IgG1 isotype [45], [46] and were generously provided by Human Genome Sciences (Rockville, MD, USA). In some experiments the cells were preincubated with a pan-caspase inhibitor, *N*-benzyloxy-carbonyl-Val-Ala-Asp(OMe)-fluoromethylketone (zVADfmk, Sigma, St Louis, USA).

### Cell cycle analysis by propidium iodide/fluorescence activated cell sorting

Cells were harvested with trypsin, washed, fixed and resuspended in 400 µl of propidium iodide (PI) solution (50 µg/ml PI, 0.1% Triton X-100, and 0.1% sodium citrate in PBS) (Cycle Plus DNA Staining Kit, Becton Dickinson, San José, CA, USA). The cells were then analyzed by flow cytometry using a software dedicated for DNA analysis (ModFit LT Software, Verity Software House, Tophsam, ME, USA).

### Western blot analysis

Whole cell extracts were obtained lysing the cells in a buffer containing 20 mM HEPES, 50 mM NaCl, 10 mM EDTA, 2 mM EGTA, 0.5% NP-40, 1 mM DTT, 0.1 mM PMSF, 2 µg/ml Leupeptin, 2 µg/ml Aprotinin, 25 mM NaF, and 10 mM Na_3_VO_4_. After incubation for 30 min on ice, the protein lysates were cleared of debris by centrifugation at 10,000 *g* for 10 min. The protein concentration in the soluble supernatant, was determined using the Bio-Rad protein assay (Bio-Rad, Richmond, VA, USA). Cellular lysates were resolved by 12% SDS-PAGE under reducing and denaturing conditions and transferred to nitrocellulose filter. The blots were blocked using 5% non-fat dry milk in TBST (10 mM Tris-HCl pH 8.0, 150 mM NaCl, 0.1% Tween 20) for 1 hour at room temperature, followed by incubation with primary antibodies overnight at 4°C. After washing with TBST, the filters were incubated with appropriate horseradish-peroxidase-conjugated secondary antibodies (Bio-Rad, CA, USA) for 1 hour at room temperature. Immunoreactivity was revealed by using an ECL detection kit (Pierce. IL, USA). The primary antibodies were anti-caspase-3 (Upstate Biotechnology, Lake Placid NY, USA), anti-caspase-8 (Upstate Biotechnology, Lake Placid NY, USA), anti-PARP (R&D System Inc., Minneapolis, MN), anti-TRAIL-R2 (Alexis Biochemicals, San Diego, CA, USA) and anti-actin (Oncogene research Products, Cambridge, MA), the last used as loading control.

### Flow cytometry analysis of TRAIL-R2

T98G and U251 cells were detached from the tissue culture flasks using a non-enzymatic detaching solution (Sigma, St Louis, USA). Cell aliquots were washed twice in cold PBS and then incubated with 5 µg/ml of PE-conjugated anti-TRAIL-R2 (R&D System, Minneapolis, USA) for 1 h at 4°C. The isotypic control antibody was mouse IgG conjugated with PE (R&D System, Minneapolis, USA). After three washes with PBS, cells were immediately analyzed for fluorescence using FAC-Scan (Becton Dickinson, San José, CA, USA).

### Analysis of mithocondrial depolarization

5,5′,6,6-tetrachloro-1,1′,3,3′-tetraethylbenzimidazzolylcarbocyanine (JC-1, Pharmingen, USA) was used to measure mitochondrial depolarization in T98G and U251 cells. Cells were treated for appropriate time with different compounds, then collected by trypsinization and incubated in complete media for 10 min. Then, the cells were incubated in 5 µg/ml JC-1 at 37°C for 15 min and washed with PBS. Both red (λem: 590 nm for FL2-H) and green (λem: 527 nm for FL1-H) fluorescence emissions were analysed by FACS at excitation wavelength of 488 nm.

### Apoptosis assessment by Annexin V staining

After drug treatments, cells were resuspended in 200 µl staining solution (containing Annexin V fluorescein and propidium iodide in a Hepes buffer, Annexin V-FITC Staining Kit, Pharmingen, San Jose, CA, USA). Following incubation at room temperature for 15 min, cells were analyzed by flow cytometry. Annexin V binds to those cells that express phosphatidylserine on the outer layer of the cell membrane, and propidium iodide stains the cellular DNA of those cells with a compromised cell membrane. This allows for the discrimination of live cells (unstained with either fluorochrome) from apoptotic cells (stained only with Annexin V) and necrotic cells (stained with both Annexin V and propidium iodide).

### Cell transfection

Transient transfections of T98G and U251 cells with small interfering (si)RNA were carried out using Lipofectamine 2000 (Invitrogen, Carlsbad, CA, USA). Two chemically synthesized siRNAs (Silencer Select Pre-designed and Validated siRNA) to TRAIL-R1 (S16764) and TRAIL-R2 (S16756), and scrambled siRNAs were purchased from Ambion and transfected at 10 nM final concentration. After 72 hours, the cells were treated with no additives (Control) or 50 ng/ml TRAIL or 10 µM Salinomycin or both drugs at the above doses. After 72 hours, the expression of TRAIL-R2 was assayed by flow cytometry.

### Xenograft assays

All animal procedures were performed according to the national Animal Experimentation guidelines (D.L.116/92) upon approval of the experimental protocol by the Institutional Animal Experimentation Committee. NSG mice were purchased from JACKSON Laboratories and maintained in microisolation cages. For the xenograft assay, U251 cells (10^6^ cells in 100 µL of saline/Matrigel (BD Pharmigen San Jose, Ca), 1∶1 v/v) were injected subcutaneously into the right flank of 6-weeks-old female animals. The size of the tumors was measured by caliper twice a week, and tumor volumes were calculated using the following formula: π/6×d^2^×D. Treatments were started after tumor reached approximately 100 mm^3^. TRAIL (5 mg/kg) and salinomycin (200 ng/kg) were administered 3 times/week, intraperitoneally. Control mice received vehicle only, according to the same schedule.

The statistical significance of the results was evaluated by ANOVA and Bonferroni post-tests. All statistical analyses were performed using GraphPad Prism v.4.0 for Windows (GraphPad Software, San Diego, CA, www.graphpad.com) and statistical significance was accepted up to 0.05. P values are displayed on the graphs using a single asterisk for significances ranging from 0.05 to 0.01, two asterisks for values between 0.001 and 0.01 and three when statistical differences produced significance below 0.001.

The presence of apoptotic cells in the tumor sections was evaluated by the TUNEL reaction, using the Boheringer Mannheim in situ Cell Detection Kit, Immunofluorescence.

### Statistical analysis

Data were analyzed using parametric statistics with one-way analysis of variance (ANOVA). *Post hoc* tests included the Student's t-Test and the Tukey multiple comparison tests as appropriate using Prism (GraphPad, San Diego, CA, USA). Data are presented as mean value ± SEM from free independent experiments. Significance was set at p<0.05.
